# The comprehensive analysis of DEG/ENaC subunits in *Hydra* reveals a large variety of peptide-gated channels, potentially involved in neuromuscular transmission

**DOI:** 10.1186/s12915-014-0084-2

**Published:** 2014-10-14

**Authors:** Marc Assmann, Anne Kuhn, Stefan Dürrnagel, Thomas W Holstein, Stefan Gründer

**Affiliations:** Department of Physiology, RWTH Aachen University, D-52074 Aachen, Germany; Molecular Evolution and Genomics, Center for Organismal Studies, D-69120 Heidelberg, Germany

**Keywords:** ASIC, DEG/ENaC, Evolution, Ligand-gated ion channel, Nervous system, Neuropeptide

## Abstract

**Background:**

It is generally the case that fast transmission at neural synapses is mediated by small molecule neurotransmitters. The simple nervous system of the cnidarian *Hydra*, however, contains a large repertoire of neuropeptides and it has been suggested that neuropeptides are the principal transmitters of *Hydra*. An ion channel directly gated by Hydra-RFamide neuropeptides has indeed been identified in *Hydra* – the Hydra Na^+^ channel (HyNaC) 2/3/5, which is expressed at the oral side of the tentacle base. Hydra-RFamides are more widely expressed, however, being found in neurons of the head and peduncle region. Here, we explore whether further peptide-gated HyNaCs exist, where in the animal they are expressed, and whether they are all gated by Hydra-RFamides.

**Results:**

We report molecular cloning of seven new HyNaC subunits – HyNaC6 to HyNaC12, all of which are members of the DEG/ENaC gene family. In *Xenopus* oocytes, these subunits assemble together with the four already known subunits into thirteen different ion channels that are directly gated by Hydra-RFamide neuropeptides with high affinity (up to 40 nM). *In situ* hybridization suggests that HyNaCs are expressed in epitheliomuscular cells at the oral and the aboral side of the tentacle base and at the peduncle. Moreover, diminazene, an inhibitor of HyNaCs, delayed tentacle movement in live *Hydra*.

**Conclusions:**

Our results show that *Hydra* has a large variety of peptide-gated ion channels that are activated by a restricted number of related neuropeptides. The existence and expression pattern of these channels, and behavioral effects induced by channel blockers, suggests that *Hydra* co-opted neuropeptides for fast neuromuscular transmission.

**Electronic supplementary material:**

The online version of this article (doi:10.1186/s12915-014-0084-2) contains supplementary material, which is available to authorized users.

## Background

In general, fast neurotransmission is mediated by small molecule neurotransmitters acting on ionotropic receptors at the post-synaptic membrane, while neuropeptides bind G-protein-coupled receptors (GPCRs) and mediate slow neuromodulatory transmission. So far, there are only two exceptions to this general pattern: the Phe-Met-Arg-Phe-amide (FMRFamide) activated Na^+^ channel (FaNaC) in snails [1,2] and the RF-amide activated Hydra Na^+^ channel (HyNaC) [3,4]. Both are ionotropic receptors, but their ligands are neuropeptides belonging to a family of FMRFamide-like peptides (FLPs) conserved from invertebrates to vertebrates [5]. Furthermore, FaNaC mediates fast responses of snail neurons to Phe-Met-Arg-Phe-amide (FMRFamide) [1,6–8], a common neuropeptide in many nervous systems [9].

FaNaC and HyNaC both belong to the degenerin/epithelial Na^+^ channel (DEG/ENaC) gene family [10] but are not species orthologs. They rather belong to different branches of the DEG/ENaC family [3,4]. DEG/ENaCs are cation channels that are blocked by the diuretic amiloride and share sequence homology and a secondary structure characterized by two transmembrane spanning domains (TMDs) and a large extracellular domain (ECD). They have various functions. Degenerins from *Caenorhabditis elegans* are mechanosensitive channels that mediate touch transduction [11]. In contrast, ENaC is a constitutively open channel that mediates Na^+^ reabsorption in the mammalian kidney [12]. Other family members from mammals are the acid-sensing ion channels (ASICs), ionotropic receptors for protons [13], and the bile-acid sensitive ion channel BASIC [14]. ASICs and BASIC are closely related to HyNaCs [3] and, interestingly, ASICs are modulated by direct binding of FLPs [15]. *Pickpocket* genes (PPKs) encode DEG/ENaCs in *Drosophila* and mediate diverse functions such as salt and water taste [16-18], mechanical nociception [19] and pheromone-sensing [20-22].

So far, four HyNaC subunits have been characterized: HyNaC2 to HyNaC5 [3,4]; *hynac1* probably is a pseudogene because it lacks an initiator methionine [3]. It is now accepted that DEG/ENaCs are trimers [23-25]; while FaNaC is a homotrimer [2], HyNaC is a heterotrimer consisting of three different subunits, HyNaC2, HyNaC3 and HyNaC5 [3,4]. *hynac2*, *hynac3* and *hynac5* are co-expressed at the base of the tentacles, most likely in epitheliomuscular cells. Hydra-RFamides are expressed in neurons from the hypostomal and the peduncle region of *Hydra* [26,27], where they are contained within large dense core vesicles in axon terminals of neurons contacting epitheliomuscular cells, suggesting that they contribute to neuromuscular transmission in *Hydra* [28,29]. Since tentacle curling is delayed by amiloride [4], it has been proposed that HyNaC is involved in movement of the tentacles [4]. While *hynac2* and *hynac3* are uniformly expressed at the tentacle base [3,4], *hynac5* is restricted to the oral side [4], suggesting that HyNaC2/3/5 is active only in epitheliomuscular cells at the oral side of the tentacle base. In contrast, *hynac4* localizes to the aboral side of the tentacles [3,4]. HyNaC4 does not assemble with HyNaC2 and HyNaC3 in a functional channel [3], suggesting the existence of further HyNaC subunits at the aboral side of the tentacles.

Since *Hydra* belongs to the phylum Cnidaria, which is a sister phylum to all bilaterians, the distant relation of FaNaC and HyNaC suggests that the common ancestor to all DEG/ENaCs might have been a peptide-gated ion channel. Alternatively, peptide-gating might have evolved twice independently in the DEG/ENaC gene family. A more definite answer to this question needs a survey of the full repertoire of DEG/ENaCs in *Hydra* to establish whether all DEG/ENaCs in *Hydra* are peptide-gated channels. Moreover, the presence of many more neuropeptides in the *Hydra* nervous system [30] and the presence of the precursors that encode Hydra-RFamides I and II [27] at the peduncle region [31] raises the questions of the existence of HyNaCs that are gated by peptides other than Hydra-RFamides I and II or that are expressed at the peduncle and activated by Hydra-RFamides I and II.

Here, we report a comprehensive analysis of the DEG/ENaC gene family in *Hydra*. We show that there is a surprising variety of HyNaCs and that, with one possible exception, HyNaCs are heterotrimers and are activated by Hydra-RFamides I and II. Some HyNaCs are expressed at the peduncle where they could mediate fast responses to RFamides I and II. Moreover, we establish the rules by which the DEG/ENaC subunits in *Hydra* assemble into functional peptide-gated channels.

## Results

### The comprehensive analysis of DEG/ENaCs in *Hydra*

**Figure 1:**
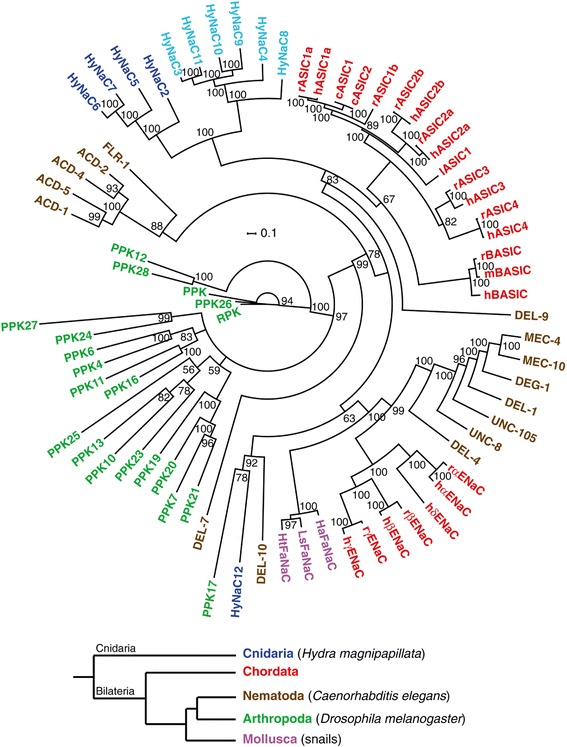
**Consensus tree for the DEG**/**ENaC family.** The phylogenetic tree was generated by Bayesian analysis with the program MrBayes 3.2 (see Methods). The numbers at nodes indicate the posterior probabilities computed by MrBayes for the respective node. Scale bar indicates amino acid exchanges per site. Colors indicate the following phyla: blue, Cnidaria; red, Chordata; green, Arthropoda; brown, Nematoda; pink, Mollusca. Abbrevations of species names are as follows: c, chicken (*Gallus gallus*); h, human (*Homo sapiens*); Ha, *Helix aspersa*; Ht, *Helisoma trivolvis*; l, lamprey (*Lampetra fluviatilis*), Ls, *Lymnaea stagnalis*; m, mouse (*Mus musculus*); r, rat (*Rattus norvegicus*). Other proteins are from *C. elegans*, *Drosophila melanogaster* or *Hydra magnipapillata*. Accession numbers are as follows: acid-sensitive degenerin (ACD)-1 [GenBank:NM_058894], ACD-2 [GenBank:NM_058892], ACD-4 [GenBank:NM_072829], ACD-5 [GenBank:NM_058795], cASIC1 [EMBL:AY956393], cASIC2 [GenBank:NM_001040467], hASIC1a [EMBL:U78180], hASIC2a [EMBL:U50352], hASIC2b [GenBank:NM_18337], hASIC3 [EMBL:AF095897], hASIC4 [EMBL:AJ271643], lASIC1 [EMBL:AAY28983], rASIC1a [EMBL:U94403], rASIC1b [EMBL:AJ309926], rASIC2a [EMBL:U53211], rASIC2b [EMBL:AB049451], rASIC3 [EMBL:AF013598], rASIC4 [EMBL:AJ271642], hBASIC [EMBL:AJ252011], mBASIC [EMBL:Y19035], rBASIC [EMBL:Y19034], DEG-1 NM_076910; degenerin-like (DEL)-1 [EMBL:U76403], DEL-4 [GenBank:NM_059829], DEL-7 [GenBank:NM_068875], DEL-9 [GenBank:NM_076221], DEL-10 [GenBank:NM-062901], αhENaC [EMBL:X76180], βhENaC [EMBL:X87159], γhENaC [EMBL:X87160], δhENaC [EMBL:U38254], αrENaC [EMBL:X70521], βrENaC [EMBL:X77932], γrENaC [EMBL:X77933], HtFaNaC [EMBL:AF254118], LsFaNaC [EMBL:AF335548], HaFaNaC [EMBL:X92113], fluoride-resistant (FLR)-1 [EMBL:AB012617], HyNaC2 [EMBL:AM393878], HyNaC3 [EMBL:AM393880], HyNaC4 [EMBL:AM393881], HyNaC5 [EMBL:FN257513], HyNaC6 [EMBL:HG422725], HyNaC7 [EMBL:HG422726], HyNaC8 [EMBL:HG422727], HyNaC9 [EMBL:HG422728], HyNaC10 [EMBL:HG422729], HyNaC11 [EMBL:HG422730], HyNaC12 [EMBL:HG422731], mechanosensitive (MEC)-4 [EMBL:X58982], MEC-10 [EMBL:L25312], pickpocket (PPK) [EMBL:Y16225], PPK4 [GenBank:NM_206137], PPK6 [GenBank:NM_137617], PPK7 [GenBank:NM_135172], PPK10 [GenBank:NM_001038805], PPK11 [GenBank:NM_001038798], PPK12 [GenBank:NM_137828], PPK13 [GenBank:NM_001014495], PPK16 [GenBank:NM_001038797], PPK17 [GenBank:NM_135965], PPK19 [EMBL:AY226547], PPK20 [GenBank:NM_143448], PPK21 [GenBank:NM_143447], PPK23 [GenBank:NM_001014749], PPK24 [GenBank:NM_143603], PPK25 [GenBank:NM_206044], PPK26 [GenBank:NM_139868], PPK27 [GenBank:NM_139569], PPK28 [GenBank:NM_001014748], ripped pocket (RPK) [EMBL:Y12640], uncoordinated (UNC)-8 [EMBL:U76402], UNC-105 [GenBank:NM_063301].

We cloned seven new cDNAs coding for proteins with high homology to the known HyNaCs (see Methods). We named these proteins HyNaC6 to HyNaC12. They have a size of 454 to 507 amino acids and a predicted molecular mass of 52.9 to 58.4 kDa. In general, the amino acid sequence identity of all HyNaCs varies between 29% and 65%. The exception is HyNaC12, which is only 14% to 17% identical to other HyNaCs. In addition, HyNaC3 and HyNaC11 are 85% identical to each other. Phylogenetic analyses revealed that HyNaC2 to HyNaC11 form a group of closely related channels within the DEG/ENaC gene family, suggesting that they derived from a single ancestor. HyNaC6 and HyNaC7 are more closely related to HyNaC2 and HyNaC5, whereas HyNaC8 to HyNaC11 are more closely related to HyNaC3 and HyNaC4, respectively, defining two subgroups within the HyNaC branch (highlighted by light and dark blue in Figure 1 and Additional file 1: Figure S1). Supporting previous results [3], HyNaC2 to HyNaC11 form a monophyletic group with ASIC and BASIC (see Figure 1 and Additional file 1: Figure S1). The grouping of HyNaCs and their relationship to ASICs and BASIC is apparent in the maximum likelihood analysis [see Additional file 1: Figure S1] and confirmed at a higher level of support by Bayesian analysis (Figure 1). The striking exception is HyNaC12, which is isolated from other HyNaCs or ASICs and BASIC (Figure 1). While the genes coding for HyNaC3 to HyNaC11 have introns, the sequence coding for HyNaC12 is present as a single uninterrupted open reading frame in the *Hydra magnipapillata* genome, showing that *hynac12* is intronless, and suggesting that it derived by retrotransposition early in cnidarian evolution. Retrotransposition is relatively common in *Hydra* [32].

The database contains the first 150 amino acids of another predicted protein that shows high homology to HyNaCs but like HyNaC1 [3], HyNaC13 lacked an initiator methionine and we were not able to clone it from *Hydra* cDNA. Therefore, we conclude that *hynac13* is an inactive pseudogene. Since extensive screening of the genomic database revealed no further DEG/ENaC homologs, we conclude that HyNaC2 to HyNaC12 constitute the whole DEG/ENaC gene family in *Hydra magnipapillata*.

**Figure 2:**
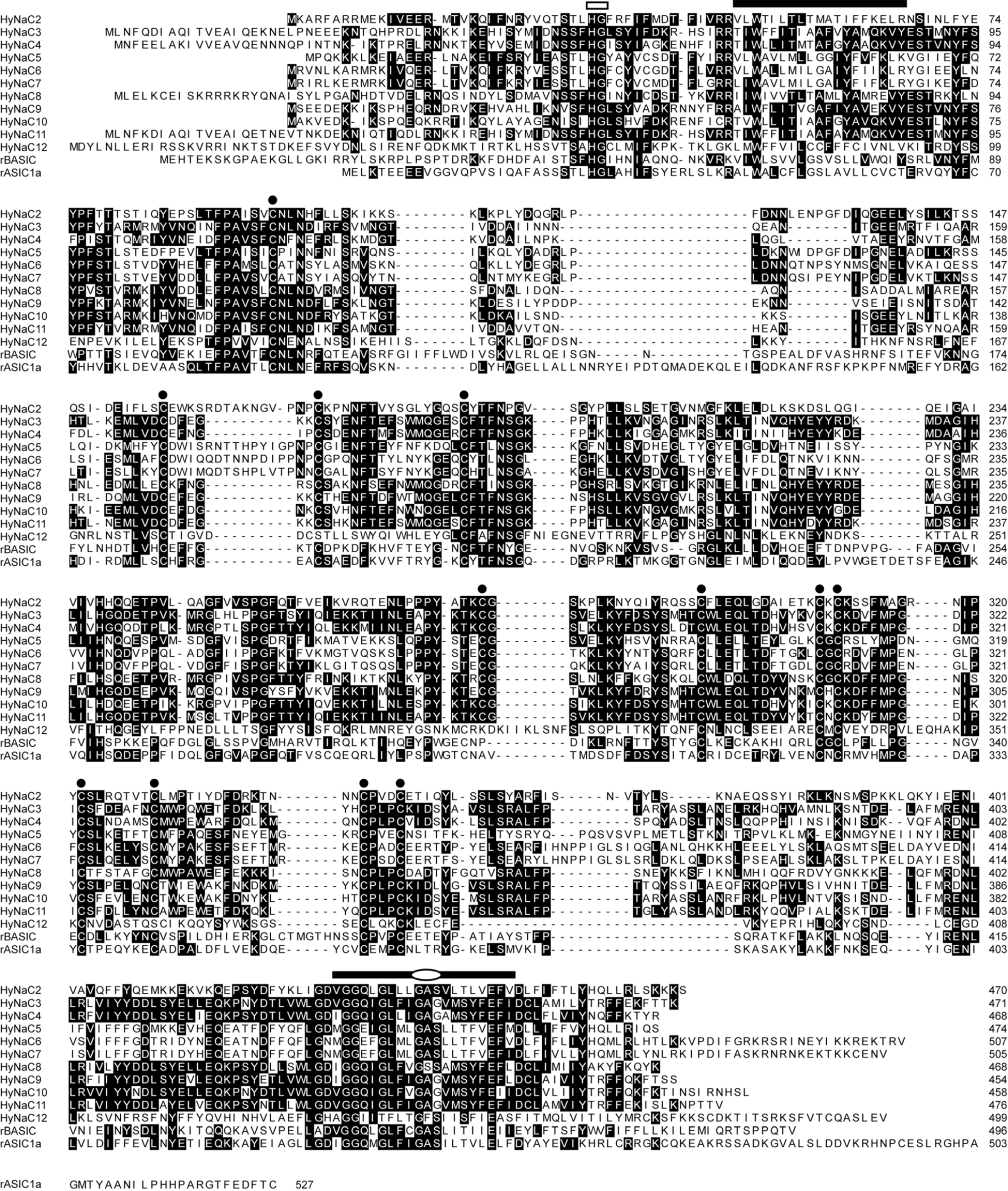
**Sequence alignment of HyNaC2 – HyNaC12 with ASIC1a and BASIC.** HyNaCs share conserved motifs and structures that are typical for DEG/ENaCs. Conserved amino acids are highlighted as white letters on black background. Bars indicate the putative position of TMDs and circles conserved cysteines in the ECD. The selectivity filter in TMD2 is indicated by an ellipse and the conserved N-terminal HG motif by an open bar. Accession numbers can be found in the legend to Figure 1. ASIC, acid-sensing ion channel; BASIC, bile-acid sensitive ion channel; ECD, extracellular domain; HyNaC, Hydra Na^+^ channel; TMDs, transmembrane domains.

All HyNaCs share conserved structures that are typical for DEG/ENaCs: they have short N- and C-termini, two TMDs and a large ECD between the TMDs [10]. Sequence motifs that are highly conserved in DEG/ENaCs are also at least partially present in HyNaCs; examples are the N-terminal HG motif [33], a di-arginine motif right before TMD1, a highly conserved tryptophane in TMD1 and the selectivity filter motif ‘GAS’ in TMD2 [34,35]. Conserved cysteine residues in the ECD [36] indicate a conserved tertiary structure (Figure 2).

### New HyNaCs contribute to a variety of functional ion channels

**Figure 3:**
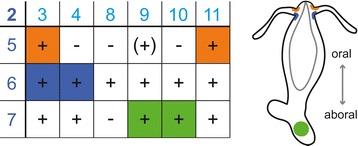
**Rules of subunit assembly.** Left, the table shows possible trimeric combinations of HyNaCs. HyNaC2 has to be present for a peptide-gated HyNaC. Subunits in the top row and the left row belong to the two subgroups defined by phylogenetic relation. A functional channel activated by Hydra-RFamides is represented by ‘ + ‘, a non-functional channel by ‘-‘. Activation by Ca^2+^ removal but insensitivity to Hydra-RFamides is indicated by ‘(+)’. Subunit combinations that are co-expressed in Hydra as revealed by ISH are shown on colored background. Orange represents expression at the oral side of the tentacle base, blue at the aboral side and green at the peduncle. Right, scheme illustrating expression sites of HyNaCs. HyNaC, Hydra Na^+^ channel; ISH, *in situ* hybridization.

We tested the function of the new HyNaCs by co-expression of different HyNaC subunit combinations in *Xenopus* oocytes. We screened for functional subunit combinations by application of 5 μM Hydra-RFamide I, a potent agonist of HyNaC2/3/5 [4]. When we expressed an individual HyNaC subunit we could never elicit currents. Similarly, the simultaneous expression of several cRNA never resulted in functional ion channels when HyNaC2 was absent. When HyNaC2 was part of the injected cRNA pool, however, Hydra-RFamide I elicited currents. Simultaneous expression of HyNaC2 with a single other HyNaC resulted only in a functional channel when we co-expressed HyNaC2 and HyNaC3; it was already known that HyNaC2 and HyNaC3 form a low-affinity peptide-gated channel [3]. Expression of HyNaC2 with two other HyNaCs, however, resulted in several functional ion channels (Figure 3).

Assembly of functional channels followed two simple rules. First, HyNaC2 had to be present. Second, one of the other two subunits had to be from subgroup 1 (dark blue in Figure 1) containing HyNaC5 to HyNaC7 and the other one from subgroup 2 (light blue in Figure 1) containing HyNaC3, HyNaC4 and HyNaC8 to HyNaC11 as defined by phylogenetic relationship (Figure 1). Specifically, HyNaC2 and HyNaC5 formed functional heteromers with HyNaC3 or HyNaC11, while HyNaC2 and HyNaC6 formed functional heteromers with all HyNaCs of the second subgroup. HyNaC2 and HyNaC7 formed functional heteromers with all members of the second subgroup except HyNaC8 (Figure 3).

**Figure 4:**
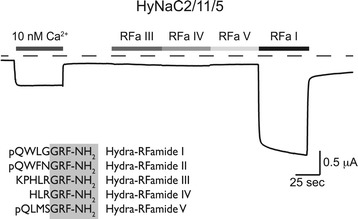
**HyNaCs are insensitive to Hydra-RFamides III to V.** Representative current trace showing activation of HyNaCs by the removal of extracellular divalent cations or by 5 μM Hydra-RFamide I. In contrast, 5 μM Hydra-RFamide III, IV or V did not elicit a current for any functional HyNaC heterotrimer; a current trace from HyNaC2/11/5 is shown as an example. Dashed line represents the zero current level. Amino acid sequences of Hydra-RFamides I to V are shown at the bottom. HyNaC, Hydra Na^+^ channel.

The contribution of the distantly related HyNaC12 to peptide-gated channels was assessed by co-expression with HyNaC2 and one or several other HyNaCs (HyNaC3 to HyNaC11). In addition, HyNaC12 was co-expressed with pools of different HyNaCs without HyNaC2. In no case did this result in channels that could be activated by Hydra-RFamide I; in the case of HyNaC2/3, current amplitude and apparent peptide affinity were not increased by co-expression of HyNaC12. We conclude that HyNaC12 does not co-assemble with other HyNaCs into channels activated by Hydra-RFamide I. Since the closely related BASIC can efficiently be opened by lowering the concentration of extracellular divalent cations [37], reduction of extracellular divalent cation concentrations provides a potential means to open HyNaCs independently of specific agonists. In fact, all HyNaCs that were activated by Hydra-RFamide I were opened by lowering the extracellular Ca^2+^ concentration ([Ca^2+^]_e_) to 10 nM (Figure 4). Moreover, HyNaC2/9/5, which was insensitive to Hydra-RFamide I, was also opened by lowering [Ca^2+^]_e_, revealing that this combination of subunits assembles into a plasma membrane expressed ion channel, which was not gated by Hydra-RFamide I. In contrast, HyNaC12, whether expressed alone or in combination with other HyNaCs, was not opened by lowering [Ca^2+^]_e_. Some DEG/ENaCs are constitutively opened by an amino acid substitution at the so-called DEG-position [38]. To further investigate whether HyNaC12 forms a functional homomeric channel, we substituted a glycine at the DEG-position by threonine (G436T), which has a large side chain. This substitution did, however, not constitutively open HyNaC12 (data not shown). This applies also for the co-expression of HyNaC12-G436T together with pools of different wild-type HyNaC subunits, leaving the function of HyNaC12 unknown.

Since Hydra-RFamide II had been shown previously to activate HyNaC2/3/5 [4], we tested Hydra-RFamides II to V for channel activation. All channels that were activated by Hydra-RFamide I were also activated by Hydra-RFamide II (data not shown). Instead Hydra-RFamide III to V (5 μM) failed to activate any functional HyNaC heterotrimer as well as HyNaCs containing HyNaC12 (Figure 4).

### Expression pattern of HyNaCs reveals putative physiological subunit combinations

**Figure 5:**
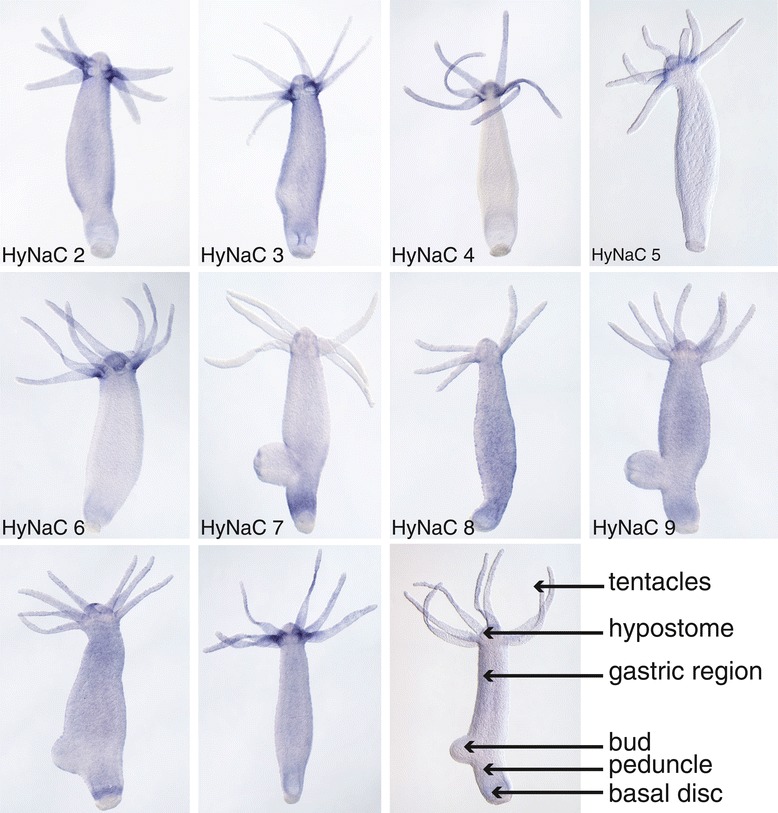
***hynacs*** **are expressed at the base of the tentacles or at the peduncle.** Whole mount *in situ* hybridizations revealed a diverse expression of different HyNaCs. *hynac2* to *hynac6* and *hynac11* are expressed at the base of the tentacles, while *hynac7* and *hynac10* are expressed at the peduncle. *hynac8* and *hynac9* are expressed ubiquitously along the whole body column. Note that *hynac2* is faintly expressed over the whole body column including the peduncle. Expression of *hynac12* could not be detected by *in situ* hybridization.

**Figure 6:**
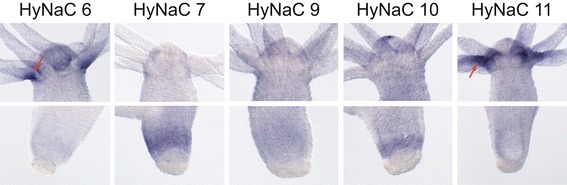
***hynacs*** **are differentially expressed at the base of the tentacles or at the peduncle.** Magnifications of the *in situ* hybridizations for *hynac6*, *hynac7* and *hynac9* to *hynac11* from Figure 5, demonstrating more precisely their expression patterns at the tentacle base and the peduncle.

The expression patterns of *hynacs* were studied by *in situ* hybridization (ISH). It had already been shown that *hynac2* to *hynac5* are expressed at the base of the tentacles of adult animals [3,4]. While *hynac2* and *hynac3* are uniformly expressed at the tentacle base, *hynac4* and *hynac*5 are asymmetrically expressed. *hynac5* is strongly expressed at the oral side of each tentacle base with a gradient toward the aboral side [4], and *hynac4* expression is restricted to the aboral side of the tentacle base [3]. Similar to *hynac4* and *hynac5*, *hynac6* and *hynac11* were asymmetrically expressed at the base of the tentacles (Figures 5 and 6). While expression of *hynac11* was restricted to the oral side, similar to *hynac5*, expression of *hynac*6 was restricted to the aboral side (Figure 6), similar to *hynac*4. These findings, together with the formation of functional heteromeric channels in oocytes, suggest the presence of HyNaC2/3/5 and HyNaC2/11/5 heterotrimers at the oral side and of HyNaC2/3/6 and HyNaC2/4/6 heterotrimers at the aboral side of the tentacle base (Figure 3). The expression pattern is typical for expression in epitheliomuscular cells (for an example of *hynac4* see Additional file 2: Figure S2).

The remaining *hynacs* were not expressed at the base of the tentacles (Figure 5). *hynac7* and *hynac10* were expressed at the peduncle (Figures 5 and 6), a region slightly above the basal disk. *hynac10* was also expressed at the hypostome (oral region) (Figure 6). *hynac8* and *hynac9* were expressed over the whole body column but *hynac9* had an increased expression level in the peduncle region (Figure 5). Since HyNaC2 was indispensable for formation of functional HyNaCs in the oocyte expression system, we re-evaluated its expression pattern and found slight expression of *hynac2* over the whole body column including the peduncle (Figure 5). These findings suggest the presence of HyNaC2/9/7 and HyNaC2/10/7 heterotrimers at the peduncle region. Since neurons expressing the preprohormone A gene that encodes the Hydra-RFamides I to IV are present at the tentacle bases, hypostome, upper gastric region and peduncle [27,31,39], it is possible that cells expressing HyNaCs in the peduncle make synaptic junctions with neurons expressing Hydra-RFamides.

We were not able to detect *hynac12* by *in situ* hybridization (Figure 5), suggesting that this subunit may be expressed in a restricted location or in low abundance. Moreover, *hynac8* expression was faint and rather diffuse and we, therefore, could not assign a putative subunit combination containing HyNaC8. Figure 3 summarizes the putative physiological subunit combinations of HyNaCs.

### HyNaCs have high-affinity for Hydra-RFamide I and II

**Figure 7:**
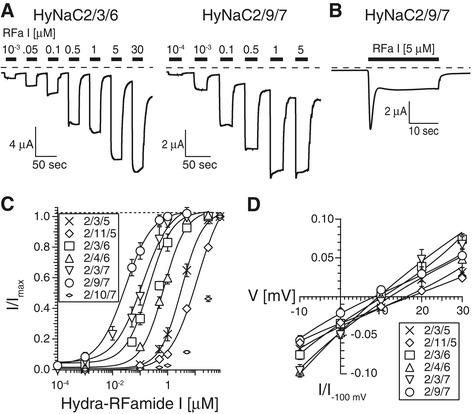
**HyNaCs are activated by Hydra-RFamide I. A)** Representative current traces showing concentration-dependent activation of HyNaC2/3/6 and HyNaC2/9/7 by Hydra-RFamide I when oocytes had been injected with EGTA. Dashed lines represent the zero current level. **B)** When oocytes had not been injected with EGTA, Hydra-RFamide I elicited biphasic currents; an oocyte expressing HyNaC2/9/7 is shown as an example. **C)** Concentration-response curves for HyNaCs and Hydra-RFamide I. Currents were normalized to the currents at the highest agonist concentration, which had amplitudes of 9.2 ± 1.9 μA (*n* = 9; 2/3/5), 2.6 ± 0.4 μA (*n* = 12; 2/11/5), 15.7 ± 3.2 μA (*n* = 8; 2/3/6), 10.1 ± 1.7 μA (*n* = 12; 2/4/6), 19.6 ± 1.8 μA (*n* = 10; 2/3/7), 7.2 ± 0.9 μA (*n* = 10; 2/9/7) and 10.6 ± 1.8 μA (*n* = 8; 2/10/7), respectively. Lines represent fits to the Hill equation. **D)** I/V curves for putative physiological HyNaCs, revealing slightly positive reversal potentials. Voltage ramps were run from −100 mV to +30 mV in two seconds. Background currents had been subtracted by voltage ramps in the absence of agonist. Currents were normalized to the current at −100 mV. EGTA, ethylene glycol tetraacetic acid; HyNaC, Hydra Na^+^ channel.

We determined for HyNaCs with an overlapping expression pattern, and hence a putative occurrence *in vivo*, apparent affinity for Hydra-RFamide I. HyNaC2/3/5 has a high Ca^2+^ permeability, which leads to activation of Ca^2+^-activated chloride channels (CaCCs) that are endogenously expressed in *Xenopus* oocytes [40]. A biphasic current with an initial peak and a slowly developing sustained phase is indicative for activation of CaCCs. All new HyNaCs had the same biphasic current as HyNaC2/3/5 (Figure 7B), suggesting high Ca^2+^ permeability. Therefore, we determined apparent affinity for Hydra-RFamide I with oocytes that had been injected with the Ca^2+^-chelator ethylene glycol tetraacetic acid (EGTA). In the presence of intracellular EGTA, Hydra-RFamide I induced simple on- and off-responses, demonstrating that under these conditions currents were not strongly contaminated by currents through CaCCs. The EC_50_ for Hydra-RFamide I varied over more than two orders of magnitude from 0.04 ± 0.01 μM (n = 9) for HyNaC2/9/7 to 13.8 ± 1.9 μM (n = 12) for HyNaC2/11/5; for comparison, HyNaC2/3/5 has an EC_50_ of approximately 5 μM [4]. For HyNaC2/10/7, the apparent affinity for Hydra-RFamide I was too low to calculate the EC_50_ precisely, since a saturating ligand concentration could not be reached (Figure 7C, Table 1).

**Table 1:** **Summary of the biophysical properties of putatively physiological HyNaCs**

**HyNaC**	**EC**_**50**_ **RFa I [μM]**	**Hill Coeff.**	**I**_**max**_ **RFa I [μA]**	**E**_**Rev**_ **[mV]**	**P**_**Ca**_ **/P**_**Na**_	**IC**_**50**_ **Dimi [μM]**	**I**_**Amil**_ **/I**
**2/3/5**	3.48 ± 0.55 (9)	1.2 ± 0.1	9.2 ± 1.9 (9)	16 ± 2 (9)	3.4	0.45 ± 0.09 (10)	0.5 ± 0.1 (10)
**2/11/5**	13.8 ± 1.9 (12)	0.9 ± 0.1	2.7 ± 0.3 (12)	17 ± 1 (8)	3.3	31.4 ± 9.4 (8)	0.7 ± 0.1 (8)
**2/3/6**	0.29 ± 0.06 (10)	1.2 ± 0.2	15.7 ± 3.2 (10)	9 ± 2 (8)	3.7	0.09 ± 0.02 (9)	0.3 ± 0.1 (9)
**2/4/6**	0.70 ± 0.08 (12)	1.3 ± 0.1	8.8 ± 1.5 (12)	14 ± 3 (9)	2.7	0.05 ± 0.02 (9)	0.3 ± 0.1 (9)
**2/3/7**	0.19 ± 0.04 (14)	1.6 ± 0.4	19.6 ± 1.8 (14)	12 ± 1 (8)	2.8	0.16 ± 0.08 (7)	0.3 ± 0.1 (7)
**2/9/7**	0.04 ± 0.01 (9)	1.5 ± 0.3	7.4 ± 0.9 (11)	7 ± 1 (10)	2.9	1.70 ± 0.58 (9)	0.3 ± 0.1 (9)
**2/10/7**	>30 (12)	n.d.	11.9 ± 1.5 (12)	n.d.	n.d.	5.40 ± 1.45 (6)	0.8 ± 0.1 (6)

Properties are summarized for HyNaCs with a putative physiological role as judged by functionality in oocytes and by co-expression as revealed by ISH. EC_50_ RFa I, concentration of Hydra-RFamide I at which half-maximal activation was achieved; I_max_ RFa I, current amplitude at maximal concentration of Hydra-RFamide I; E_Rev_, reversal potential of RFamide I-activated currents with standard bath; P_Ca_/P_Na_ relative permeability of Ca^2+^ to Na^+^; IC_50_ Dimi, concentration of diminazene at which half-maximal inhibition was achieved; I_Amil_/I, relative current remaining after block by 100 μM amiloride. Values indicate mean ± standard error; the number *n* of individual oocytes is indicated in brackets. HyNaC, Hydra Na^+^ channel; ISH, *in situ* hybridization.

**Figure 8:**
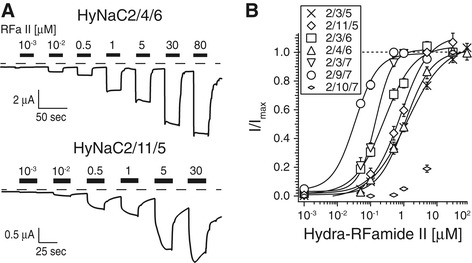
**HyNaCs are activated by Hydra-RFamide II. A)** Representative current traces showing concentration-dependent activation of HyNaC2/4/6 and HyNaC2/11/5 by Hydra-RFamide II; oocytes had been injected with EGTA. Dashed lines represent the zero current level. **B)** Concentration-response curves for HyNaCs and Hydra-RFamide II. Currents were normalized to the currents at highest agonist concentration, which had amplitudes of 5.3 ± 1.0 μA (*n* = 14; 2/3/5), 2.2 ± 0.4 μA (*n* = 7; 2/11/5), 9.2 ± 1.7 μA (*n* = 8; 2/3/6), 4.7 ± 0.8 μA (*n* = 12; 2/4/6), 11.8 ± 4.0 μA (*n* = 8; 2/3/7), 3.6 ± 1.3 μA (*n* = 8; 2/9/7) and 1.1 ± 0.4 μA (*n* = 7; 2/10/7), respectively. Lines represent fits to the Hill equation. EGTA, ethylene glycol tetraacetic acid; HyNaC, Hydra Na^+^ channel.

Previously, it had been shown that HyNaC2/3/5 has a ten times higher apparent affinity for Hydra-RFamide II than for Hydra-RFamide I [4]. We re-assessed apparent affinity of HyNaC2/3/5 for Hydra-RFamide II with EGTA-injected oocytes and found it to be 1.61 ± 0.27 μM (n = 14), slightly lower than reported for oocytes not injected with EGTA (0.34 ± 0.08 μM) [4]. Regarding the other putative physiological HyNaC heterotrimers, the EC_50_ for Hydra-RFamide II varied between 0.04 ± 0.01 μM (n = 8) for HyNaC2/9/7 and 0.95 ± 0.16 (n = 7) for HyNaC2/11/5 (Figure 8), which is in the same range as for Hydra-RFamide I. Only for HyNaC2/11/5 was the apparent affinity for Hydra-RFamide II about 15-fold higher than for Hydra-RFamide I (0.95 μM compared with 13.8 μM).

**Figure 9:**
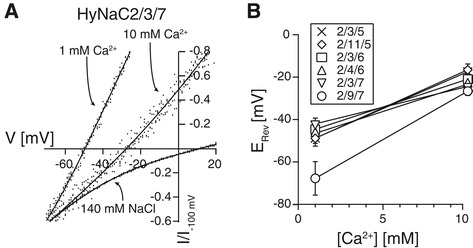
**Change in extracellular Ca** ^**2+**^ **concentration shifts the reversal potential of HyNaCs. A)** *Xenopus* oocytes expressing HyNaC2/3/7 were activated by 100 nM Hydra-RFamide I, corresponding to its EC_50_. The conductive cation in the extracellular solution was either 1 mM Ca^2+^, or 10 mM Ca^2+^, or 140 mM Na^+^. Reversal potentials were determined by voltage ramps from −100 mV to +30 mV in two seconds. Background currents had been subtracted by voltage ramps in the absence of agonist. **B)** Diagram showing the shift of the reversal potentials for different HyNaC combinations, when the extracellular solution contained 1 or 10 mM Ca^2+^ as the only conductive ion. HyNaCs had been activated by a concentration of Hydra-RFamide I that corresponds to the individual EC_50_. HyNaCs, Hydra Na^+^ channels.

Similar to HyNaC2/3/5 [4], all HyNaCs had a slightly positive reversal potential E_rev_ in standard bath (E_rev_ varied between 7.4 ± 0.5 mV, n = 10, for HyNaC2/9/7 and 17.3 ± 1.1 mV, n = 8, for HyNaC2/11/5; Figure 7D, Table 1), suggesting that they are unselective cation channels. HyNaC 2/3/5 is characterized by a high Ca^2+^ permeability with a permeability ratio P_Ca_/P_Na_ = 3.85 [40]. We investigated the Ca^2+^ permeability of the new HyNaCs by replacing extracellular Na^+^ by Ca^2+^. An increase in extracellular Ca^2+^ from 1 to 10 mM resulted in a strong rightward shift of E_rev_ (Figure 9), confirming Ca^2+^ permeability of HyNaCs. The shift was in the same range as for HyNaC2/3/5 [40], suggesting a comparable Ca^2+^ permeability. We used E_rev_ at 10 mM Ca^2+^ and at 140 mM Na^+^ as the only permeant cations (see Methods) to calculate the relative permeability P_Ca_/P_Na_ for the new HyNaCs. P_Ca_/P_Na_ ranged from 2.66 for HyNaC2/4/6 (n = 9) to 3.72 for HyNaC2/3/6 (n = 9) (Table 1), showing that all HyNaCs are highly Ca^2+^ permeable.

### Diminazene is a potent inhibitor of HyNaCs and inhibits the feeding reaction

**Figure 10:**
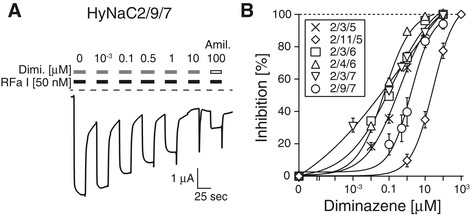
**Diminazene is a potent inhibitor of HyNaCs. A)** Current trace illustrating the concentration-dependent inhibition of HyNaC2/9/7 by diminazene. Block by 100 μM amiloride was used to compare the affinity for diminazene and amiloride. Note that current amplitude transiently increased after washout of the blockers. Dashed lines represent the zero current level. **B)** Concentration-response curves showing the concentration-dependent inhibition of HyNaCs by diminazene. HyNaCs, Hydra Na^+^ channels.

One of the hallmarks of DEG/ENaCs is the block by the diuretic amiloride [10]. Since the apparent amiloride affinity of HyNaC2/3/5 is quite low with an IC_50_ of approximately 120 μM [4], amiloride is of limited utility for the study of HyNaCs. Recently, diminazene and related compounds have been reported to potently inhibit ASICs and BASIC but not ENaC [41,42]. Since ASICs and BASIC are closely related to HyNaCs, we asked whether diminazene also inhibits HyNaCs. For most HyNaCs, 10 μM of diminazene indeed almost completely blocked HyNaC currents. The IC_50_ for diminazene varied between 0.05 ± 0.02 μM (n =9) for HyNaC2/4/6 and 1.70 ± 0.58 μM (n =9) for HyNaC2/9/7. Only HyNaC2/11/5 was blocked by diminazene with a low apparent affinity and an IC_50_ of 31.4 ± 9.4 μM (n =8). Although we did not determine apparent amiloride affinity for all HyNaCs, based on a 20% to 70% inhibition of currents by 100 μM amiloride (Figure 10, Table 1) one can estimate that diminazene has a >100-fold higher potency than amiloride for most HyNaCs.

**Figure 11:**
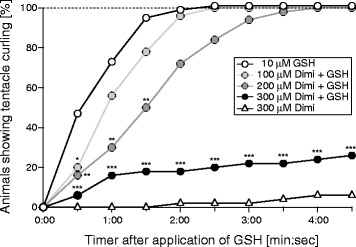
**Diminazene inhibits the feeding reaction of** ***Hydra magnipapillata*** **.** Adult animals were held in plain medium or in medium containing 100, 200 or 300 μM diminazene (Dimi). The feeding response was induced by application of 10 μM glutathione (GSH). The number of animals showing complete tentacle curling was recorded every 30 seconds. *, *P* <0.05; **, *P* <0.01; ***, *P* <0.001.

It has previously been shown that 100 μM amiloride delays the feeding reaction of living *Hydra* [4], supporting the hypothesis that amiloride-sensitive channels are involved in the feeding reaction. To find further evidence that amiloride inhibited the feeding reaction via inhibition of HyNaCs, we repeated these experiments with diminazene. Addition of glutathione (GSH; 10 μM final concentration) induced tentacle contractions and animals moved the tentacles to their mouth. After about two minutes, 100% of animals had their tentacles completely curled. When animals were held in a medium containing 100 μM diminazene, tentacle curling was slightly delayed. Increasing the concentration of diminazene to 200 μM, more strongly and significantly (*P* ≤0.01) delayed the initiation of tentacles movement such that tentacles were completely curled only after three minutes. When the concentration of diminazene was further increased to 300 μM, it strongly inhibited the feeding reaction such that even after four minutes only 20% of the animals had their tentacles completely curled (*P* ≤0.001; Figure 11). Although the potency of diminazene *in vivo* was low, it should be taken into account that septate junctions provide a significant paracellular permeability barrier to the mesogleal space of *Hydra* [43]. Moreover, it appears that diminazene requires active transport mechanisms for cellular uptake [44,45], probably strongly limiting its transcellular transport; amiloride in contrast is, in part, uncharged at neutral pH and has a significant membrane permeability [46]. It is, therefore, conceivable that diminazene reached significantly lower concentrations in the mesogleal space of *Hydra* than in the bath. Therefore, these results support the idea that HyNaCs are involved in the feeding reaction of *Hydra*.

## Discussion

### DEG/ENaCs in *Hydra* are peptide-gated

In this study, we characterized the complete gene set of ten DEG/ENaC genes in the model cnidarian *Hydra*. We found genomic evidence for two further transcribed genes, that is, *hynac1* and *hynac12*. Our data indicate, however, that these are pseudogenes that probably arose by retrotransposition from a common precursor with other HyNaCs [32]. The remaining ten genes give rise to ten HyNaC subunits that assemble in a variety of combinations into heteromeric ion channels.

HyNaCs are more closely related to each other than to any other DEG/ENaC, forming a sub-branch on the phylogenetic tree in Figure 1. Closest relatives are ASICs [13] and BASIC [14]. HyNaCs form two lower order subgroups within the HyNaC branch (Figure 1). Subgroup 1 (dark blue in Figure 1) contains HyNaC2, HyNaC5, HyNaC6 and HyNaC7, while subgroup 2 (light blue in Figure 1) contains HyNaC3, HyNaC4 and HyNaC8 to HyNaC11. Combinatorial expression of individual HyNaCs revealed the rules for subunit assembly. HyNaC2 was present in all functional channels. The second subunit had to be from subgroup 2 and the third was another subunit from subgroup 1, closely related to HyNaC2. HyNaC2/3 is the only HyNaC that contains only two different subunits yet is functional [3]. The presence of a HyNaC2-like subunit in all other channels makes 2/2/3 (and not 2/3/3) the likely composition of HyNaC2/3.

According to the rules described above, HyNaCs could theoretically assemble in 18 different combinations (Figure 3). We found that 13 of these combinations are activated by Hydra-RFamides I and II (Figure 3). One other combination (HyNaC2/9/5) was not activated by Hydra-RFamides I and II but by removing extracellular divalent cations, indicating correctly assembled plasma membrane expressed channels. It is therefore possible that HyNaC2/9/5 is activated by another neuropeptide than RFamides. Since the other four theoretical subunit combinations could neither be activated by RFamide neuropeptides nor by removal of divalent cations, it is likely that these combinations do not assemble in functional ion channels; at least not in the oocyte expression system. With the exception of HyNaC2/10/7, the functional peptide-activated channels all had high apparent ligand affinity; for some, such as HyNaC2/9/7, nanomolar concentrations of Hydra-RFamides I and II elicited robust currents (Figure 7). The high affinity for RFamides I and II renders it highly unlikely that other neuropeptides also activate HyNaCs with similar affinity. We, therefore, conclude that the physiological ligands of HyNaCs are Hydra-RFamides I and II, with the possible exceptions of HyNaC2/9/5 and HyNaC2/10/7. This conclusion lends support to the hypothesis that the common ancestor of DEG/ENaCs was a peptide-gated channel. A definite answer to this question, however, requires the analysis of DEG/ENaCs from more non-bilaterian animals.

### HyNaCs may contribute to neuromuscular transmission in *Hydra*

For six of the thirteen heterotrimers, which are activated by RFamides in the oocyte expression system, the three subunits are co-expressed (Figure 5). It is therefore likely that these six heterotrimers form peptide-gated channels *in situ*. Due to the limited sensitivity of ISH, we cannot exclude that further combinations exist *in situ*. In particular, we could not assign the locus of expression of HyNaC8. In summary, our results suggest that two different HyNaCs are present at the oral (HyNaC2/3/5 and HyNaC2/11/5) and two at the aboral side of the tentacle base (HyNaC2/3/6 and HyNaC2/4/6), respectively. Two other HyNaCs are expressed at the peduncle (HyNaC2/9/7 and HyNaC2/10/7). Since these channels are all activated by Hydra-RFamides I and II, this variety is unexpected. We speculate that the variety of HyNaCs helps to fine tune ligand affinity and perhaps subcellular location of RFamide-gated channels.

What is the function of HyNaCs? ISH from this and previous [3,4] studies suggests that HyNaCs are expressed in epitheliomuscular cells, which function as muscle cells. Since Hydra-RFamides are contained within large dense core vesicles in axon terminals of neurons contacting epitheliomuscular cells [28,29], our results identify HyNaCs as a candidate post-synaptic receptor for Hydra-RFamides, which could depolarize muscle cells to induce contractions. In agreement with this speculation, amiloride [4] and diminazene, blockers of HyNaCs, significantly delayed the feeding reaction of *Hydra* (Figure 11). In vertebrates and arthropods, however, neuromuscular transmission uses the small molecule transmitters, acetylcholine and glutamate, respectively. Sequencing of the *Hydra* genome indeed revealed seventeen genes coding for nicotinic acetylcholine receptor subunits but other key components of the vertebrate neuromuscular junction are not present in *Hydra* and the authors concluded that a canonical bilaterian neuromuscular junction was not present in the last common ancestor of cnidarians and bilaterians [32]. Moreover, it has recently been suggested that striated muscles evolved convergently in cnidarians and bilaterians from cells with ancient contractile machinery [47]. Assuming that the junction with neurons of striated muscle cells evolved after or at the same time as the evolution of striated muscles, this finding would explain why cnidarian and bilaterian muscles might use different transmitters at their junction with neurons. In summary, our results suggest that Hydra-RFamides I and II and HyNaCs should be considered as a transmitter-receptor pair mediating fast neuromuscular transmission in *Hydra*.

One feature of HyNaCs that is unique for DEG/ENaCs is their high Ca^2+^ permeability. If HyNaCs indeed carried an excitatory post-synaptic current in muscle cells then Ca^2+^ flux through HyNaCs could directly induce muscle contractions independent of release from intracellular stores. A high Ca^2+^ permeability might also confer an advantage in a freshwater animal like *Hydra* with a low extracellular Na^+^ concentration. In *Hydra littoralis* (a member of the *H. vulgaris* group, [48]) an extracellular osmolality of 16.8 mM/kg has been measured [49], significantly higher than in the surrounding freshwater but much lower than in most other animals. Thus, Ca^2+^ may carry a significant fraction of the excitatory current in the freshwater animal *Hydra*.

## Conclusions

Our study identifies in *Hydra* a variety of ion channels gated by Hydra-RFamide neuropeptides. Their expression in epithelial cells at the base of the tentacles and in the peduncle suggests a role for these channels in neuromuscular transmission. Thus, our results support the hypothesis that *Hydra* uses neuropeptides for fast neuromuscular transmission.

## Methods

### Cloning of new HyNaCs

Using the DNA sequences of HyNaC2 to HyNaC5, we performed a BLAST search against the genomic database of *Hydra magnipapillata* at the National Center for Biotechnology Information and identified the full-length protein sequence for three new HyNaCs; these HyNaCs were later named HyNaC6, HyNaC9 and HyNaC10, respectively. The complete coding sequences of these three HyNaCs were directly amplified by PCR with specific primers using cDNA extracted from adult one-day starved budding stage animals of *Hydra magnipapillata* strain 105. Other genomic sequences with homology to HyNaCs were used to design primers for 3′- and 5′-RACE. Sequences obtained by RACE-PCRs were assembled to full-length sequences, which were then amplified by PCR with specific primers using *Hydra* cDNA. The new HyNaC sequences were submitted to the EMBL-EBI-database with accession numbers HyNaC6 [EMBL:HG422725], HyNaC7 [EMBL:HG422726], HyNaC8 [EMBL:HG422727], HyNaC9 [EMBL:HG422728], HyNaC10 [EMBL:HG422729], HyNaC11 [EMBL:HG422730], HyNaC12 [EMBL:HG422731].

### Analysis of phylogenetic relationship of HyNaCs

To analyze the phyologenetic relationship of HyNaC subunits, their sequences were aligned with the sequences of other DEG/ENaC channels using ClustalX (v.2.1); highly divergent sequences at the N- and C-terminus as well as in the extracellular loop were deleted to improve the alignment by automated 1 method of trimAl (v.1.3). The best model for amino acid substitution was determined with ProtTest 3 [50]. Phylogenetic trees were established by neighbor-joining analysis with ClustalX (v.2.1), maximum likelihood analysis with PhyML (v.3.0) [51] and Bayesian analysis with MrBayes (v.3.2). For maximum likelihood analysis we used the WAG model of protein evolution and the NNI tree topology search option; bootstrap support values were obtained from 100 bootstrap samples. During Bayesian analysis an average standard deviation ≤0.0001 was estimated. Therefore we ran 10^7^ generations with one chain without Metropolis coupling. After a burn-in of 10^5^ generations every 1,000th tree was sampled. Regarding the phylogenetic relations of HyNaCs, ASICs and BASICs, all three phylogenetic analyses revealed comparable results. Relations of other DEG/ENaCs were slightly different. A ClustalW analysis of all HyNaC subunits, rASIC1a, and rBASIC was performed with the software DNASTAR (v.10.0.1; Madison, WI, USA) to draw the alignment of Figure 2.

### *In situ* hybridization

Expression patterns of HyNaCs were analyzed by whole mount *in situ* hybridization [52]. For all new HyNaCs, probes for *in situ* hybridization were subcloned into pBluescript KS; they had lengths between 600 and 1,400 bp, depending on the staining efficiency. Probes were detected with an antibody conjugated to an alkaline phosphatase and BMP Purple as substrate.

### Electrophysiology

To study the biophysical properties of the new HyNaCs, cRNA of HyNaCs was synthesized *in vitro* (mMessage mMachine kit, Ambion, Austin, Texas, USA) and injected into *Xenopus laevis* oocytes of stage V and VI. After injection, oocytes were incubated for one to two days at 19°C in oocyte Ringer’s solution 2 (OR-2), which contained (in mM): 82.5 NaCl, 2.5 KCl, 1.0 Na_2_HPO_4_, 1.0 MgCl_2_, 1.0 CaCl_2_, 5.0 HEPES, 0.5 g/l PVP, 1,000 U/l penicillin and 10 mg/l streptomycin, NaOH was used to adjust pH to 7.3. Whole cell currents were recorded from defolliculated oocytes by two-electrode voltage-clamp (TEVC) using the amplifier TurboTec 03X (npi electronic GmbH, Tamm, Germany). Standard bath solution (in mM: 140 NaCl, 10 HEPES, 1.8 CaCl_2_ and 1.0 MgCl_2_. NaOH was used to adjust pH to 7.4.) was exchanged by a pump driven system combined with the oocyte testing carousel (OTC), which was controlled by the interface OTC-20 [53]. To control the OTC-20 and to record the data to the hard drive, the software CellWorks (version 5.1.1; npi electronic GmbH, Tamm, Germany) was used. Data were sampled at 0.1 to 1 kHz and filtered at 20 Hz. HyNaCs are permeable for Ca^2+^, activating an endogenous CaCC [40]. Therefore, unless otherwise indicated, oocytes were injected with 50 nl of 20 mM EGTA 30 to 180 minutes before measurements. Reversal potentials and Ca^2+^ permeabilities of HyNaCs were studied as described previously [40].

### Data analysis

EC_50_ values for Hydra-RFamides I and II as well as IC_50_ values for diminazene were determined by fits to the Hill equation using IGOR Pro (version 6.06, WaveMetrics, Inc.). Reversal potentials were defined as the first recorded data point, where currents reversed signs from negative to positive. For I/V relationships, voltage ramps in the absence of the agonist were used to subtract background conductances. Results are reported as mean ± standard error.

## Additional files

Additional file 1
**Figure S1.** Phylogenetic tree for the DEG/ENaC family generated by maximum likelihood analysis. The phylogenetic tree was generated by maximum likelihood analysis with PhyML (see Methods). Support values are indicated. Scale bar indicates amino acid exchanges per site. Color code, abbrevations of species names, and accession numbers are as in Figure 1. Additional file 2
**Figure S2.** High magnification of whole mount in situ hybridization for *hynac4* revealing expression in epitheliomuscular cells at the base of the tentacles.

## Abbreviations

ASIC: acid-sensing ion channel
BASIC: bile acid-sensitive ion channel
CaCCs: Ca^+^-activated chloride channels
DEG: degenerin
ECD: extracellular domain
EGTA: ethylene glycol tetraacetic acid
ENaC: epithelial Na^+^ channel
FaNaC: FMRFamide-gated Na^+^ channel
HyNaC: Hydra Na^+^ channel
ISH: *in situ* hybridization
PPK: pickpocket
TMD: transmembrane domain

## References

[1] Cottrell GA, Green KA, Davies NW (1990). The neuropeptide Phe-Met-Arg-Phe-NH2 (FMRFamide) can activate a ligand-gated ion channel in Helix neurones. Pflugers Arch.

[2] Lingueglia E, Champigny G, Lazdunski M, Barbry P (1995). Cloning of the amiloride-sensitive FMRFamide peptide-gated sodium channel. Nature.

[3] Golubovic A, Kuhn A, Williamson M, Kalbacher H, Holstein TW, Grimmelikhuijzen CJ, Gründer S (2007). A peptide-gated ion channel from the freshwater polyp Hydra. J Biol Chem.

[4] Dürrnagel S, Kuhn A, Tsiairis CD, Williamson M, Kalbacher H, Grimmelikhuijzen CJ, Holstein TW, Gründer S (2010). Three homologous subunits form a high affinity peptide-gated ion channel in Hydra. J Biol Chem.

[5] Jékely G (2013). Global view of the evolution and diversity of metazoan neuropeptide signaling. Proc Natl Acad Sci U S A.

[6] Green KA, Falconer SW, Cottrell GA (1994). The neuropeptide Phe-Met-Arg-Phe-NH2 (FMRFamide) directly gates two ion channels in an identified Helix neurone. Pflugers Arch.

[7] Davey F, Harris SJ, Cottrell GA (2001). Histochemical localisation of FMRFamide-gated Na + channels in *Helisoma triolvis* and *Helix aspersa* neurones. J Neurocytol.

[8] Perry SJ, Straub VA, Schofield MG, Burke JF, Benjamin PR (2001). Neuronal expression of an FMRFamide-gated Na + channel and its modulation by acid pH. J Neurosci.

[9] Walker RJ, Papaioannou S, Holden-Dye L (2009). A review of FMRFamide- and RFamide-like peptides in metazoa. Invert Neurosci.

[10] Kellenberger S, Schild L (2002). Epithelial sodium channel/degenerin family of ion channels: a variety of functions for a shared structure. Physiol Rev.

[11] O’Hagan R, Chalfie M, Goodman MB (2005). The MEC-4 DEG/ENaC channel of caenorhabditis elegans touch receptor neurons transduces mechanical signals. Nat Neurosci.

[12] Canessa CM, Schild L, Buell G, Thorens B, Gautschi I, Horisberger JD, Rossier BC (1994). Amiloride-sensitive epithelial Na + channel is made of three homologous subunits. Nature.

[13] Waldmann R, Champigny G, Bassilana F, Heurteaux C, Lazdunski M (1997). A proton-gated cation channel involved in acid-sensing. Nature.

[14] Wiemuth D, Sahin H, Falkenburger BH, Lefevre CM, Wasmuth HE, Gründer S (2012). BASIC–a bile acid-sensitive ion channel highly expressed in bile ducts. FASEB J.

[15] Askwith CC, Cheng C, Ikuma M, Benson C, Price MP, Welsh MJ (2000). Neuropeptide FF and FMRFamide potentiate acid-evoked currents from sensory neurons and proton-gated DEG/ENaC channels. Neuron.

[16] Liu L, Leonard AS, Motto DG, Feller MA, Price MP, Johnson WA, Welsh MJ (2003). Contribution of drosophila DEG/ENaC genes to salt taste. Neuron.

[17] Cameron P, Hiroi M, Ngai J, Scott K (2010). The molecular basis for water taste in Drosophila. Nature.

[18] Chen Z, Wang Q, Wang Z (2010). The amiloride-sensitive epithelial Na + channel PPK28 is essential for drosophila gustatory water reception. J Neurosci.

[19] Zhong L, Hwang RY, Tracey WD (2010). Pickpocket is a DEG/ENaC protein required for mechanical nociception in Drosophila larvae. Curr Biol.

[20] Starostina E, Liu T, Vijayan V, Zheng Z, Siwicki KK, Pikielny CW (2012). A Drosophila DEG/ENaC subunit functions specifically in gustatory neurons required for male courtship behavior. J Neurosci.

[21] Thistle R, Cameron P, Ghorayshi A, Dennison L, Scott K (2012). Contact chemoreceptors mediate male-male repulsion and male–female attraction during Drosophila courtship. Cell.

[22] Liu T, Starostina E, Vijayan V, Pikielny CW (2012). Two Drosophila DEG/ENaC channel subunits have distinct functions in gustatory neurons that activate male courtship. J Neurosci.

[23] Jasti J, Furukawa H, Gonzales EB, Gouaux E (2007). Structure of acid-sensing ion channel 1 at 1.9 a resolution and low pH. Nature.

[24] Gonzales EB, Kawate T, Gouaux E (2009). Pore architecture and ion sites in acid-sensing ion channels and P2X receptors. Nature.

[25] Bartoi T, Augustinowski K, Polleichtner G, Gründer S, Ulbrich MH (2014). Acid-sensing ion channel (ASIC) 1a/2a heteromers have a flexible 2:1/1:2 stoichiometry. Proc Natl Acad Sci U S A.

[26] Moosler A, Rinehart KL, Grimmelikhuijzen CJ (1996). Isolation of four novel neuropeptides, the hydra-RFamides I-IV, from Hydra magnipapillata. Biochem Biophys Res Commun.

[27] Darmer D, Hauser F, Nothacker HP, Bosch TC, Williamson M, Grimmelikhuijzen CJ (1998). Three different prohormones yield a variety of Hydra-RFamide (Arg-Phe-NH2) neuropeptides in Hydra magnipapillata. Biochem J.

[28] Koizumi O, Wilson JD, Grimmelikhuijzen CJ, Westfall JA (1989). Ultrastructural localization of RFamide-like peptides in neuronal dense-cored vesicles in the peduncle of Hydra. J Exp Zool.

[29] Westfall JA (1996). Ultrastructure of synapses in the first-evolved nervous systems. J Neurocytol.

[30] Grimmelikhuijzen CJ, Leviev I, Carstensen K (1996). Peptides in the nervous systems of cnidarians: structure, function, and biosynthesis. Int Rev Cytol.

[31] Hansen GN, Williamson M, Grimmelikhuijzen CJ (2000). Two-color double-labeling in situ hybridization of whole-mount Hydra using RNA probes for five different Hydra neuropeptide preprohormones: evidence for colocalization. Cell Tissue Res.

[32] Chapman JA, Kirkness EF, Simakov O, Hampson SE, Mitros T, Weinmaier T, Rattei T, Balasubramanian PG, Borman J, Busam D, Disbennett K, Pfannkoch C, Sumin N, Sutton GG, Viswanathan LD, Walenz B, Goodstein DM, Hellsten U, Kawashima T, Prochnik SE, Putnam NH, Shu S, Blumberg B, Dana CE, Gee L, Kibler DF, Law L, Lindgens D, Martinez DE, Peng J (2010). The dynamic genome of Hydra. Nature.

[33] Gründer S, Jaeger NF, Gautschi I, Schild L, Rossier BC (1999). Identification of a highly conserved sequence at the N-terminus of the epithelial Na + channel alpha subunit involved in gating. Pflugers Arch.

[34] Kellenberger S, Gautschi I, Schild L (1999). A single point mutation in the pore region of the epithelial Na + channel changes ion selectivity by modifying molecular sieving. Proc Natl Acad Sci U S A.

[35] Snyder PM, Olson DR, Bucher DB (1999). A pore segment in DEG/ENaC Na(+) channels. J Biol Chem.

[36] Firsov D, Robert-Nicoud M, Gründer S, Schild L, Rossier BC (1999). Mutational analysis of cysteine-rich domains of the epithelium sodium channel (ENaC). Identification of cysteines essential for channel expression at the cell surface. J Biol Chem.

[37] Wiemuth D, Gründer S (2010). A single amino acid tunes Ca2+ inhibition of brain liver intestine Na + channel (BLINaC). J Biol Chem.

[38] Driscoll M, Chalfie M (1991). The mec-4 gene is a member of a family of Caenorhabditis elegans genes that can mutate to induce neuronal degeneration. Nature.

[39] Grimmelikhuijzen CJ (1985). Antisera to the sequence Arg-Phe-amide visualize neuronal centralization in hydroid polyps. Cell Tissue Res.

[40] Dürrnagel S, Falkenburger BH, Gründer S (2012). High Ca(2+) permeability of a peptide-gated DEG/ENaC from Hydra. J Gen Physiol.

[41] Chen X, Qiu L, Li M, Dürrnagel S, Orser BA, Xiong ZG, MacDonald JF (2010). Diarylamidines: high potency inhibitors of acid-sensing ion channels. Neuropharmacology.

[42] Wiemuth D, Gründer S (2011). The pharmacological profile of brain liver intestine Na + channel: inhibition by diarylamidines and activation by fenamates. Mol Pharmacol.

[43] Filshie BK, Flower NE (1977). Junctional structures in hydra. J Cell Sci.

[44] Teka IA, Kazibwe AJ, El-Sabbagh N, Al-Salabi MI, Ward CP, Eze AA, Munday JC, Maser P, Matovu E, Barrett MP, de Koning HP (2011). The diamidine diminazene aceturate is a substrate for the high-affinity pentamidine transporter: implications for the development of high resistance levels in trypanosomes. Mol Pharmacol.

[45] Ward CP, Wong PE, Burchmore RJ, de Koning HP, Barrett MP (2011). Trypanocidal furamidine analogues: influence of pyridine nitrogens on trypanocidal activity, transport kinetics, and resistance patterns. Antimicrob Agents Chemother.

[46] Benos DJ, Reyes J, Shoemaker DG (1983). Amiloride fluxes across erythrocyte membranes. Biochim Biophys Acta.

[47] Steinmetz PR, Kraus JE, Larroux C, Hammel JU, Amon-Hassenzahl A, Houliston E, Worheide G, Nickel M, Degnan BM, Technau U (2012). Independent evolution of striated muscles in cnidarians and bilaterians. Nature.

[48] Martinez DE, Iniguez AR, Percell KM, Willner JB, Signorovitch J, Campbell RD (2010). Phylogeny and biogeography of Hydra (Cnidaria: Hydridae) using mitochondrial and nuclear DNA sequences. Mol Phylogenet Evol.

[49] Benos DJ, Prusch RD (1972). Osmoregulation in fresh-water Hydra. Comp Biochem Physiol A.

[50] Darriba D, Taboada GL, Doallo R, Posada D (2011). ProtTest 3: fast selection of best-fit models of protein evolution. Bioinformatics.

[51] Guindon S, Dufayard JF, Lefort V, Anisimova M, Hordijk W, Gascuel O (2010). New algorithms and methods to estimate maximum-likelihood phylogenies: assessing the performance of PhyML 3.0. Syst Biol.

[52] Guder C, Pinho S, Nacak TG, Schmidt HA, Hobmayer B, Niehrs C, Holstein TW (2006). An ancient Wnt-Dickkopf antagonism in Hydra. Development.

[53] Madeja M, Musshoff U, Speckmann EJ (1995). Improvement and testing of a concentration-clamp system for oocytes of Xenopus laevis. J Neurosci Methods.

