# Supplementary figures and images for: The comprehensive analysis of DEG/ENaC subunits in Hydra reveals a large variety of peptide-gated channels, potentially involved in neuromuscular transmission
Source: BMC Biol. 2014 Oct 14;12:84. doi: 10.1186/s12915-014-0084-2 (PMC4212090; doi:10.1186/s12915-014-0084-2)

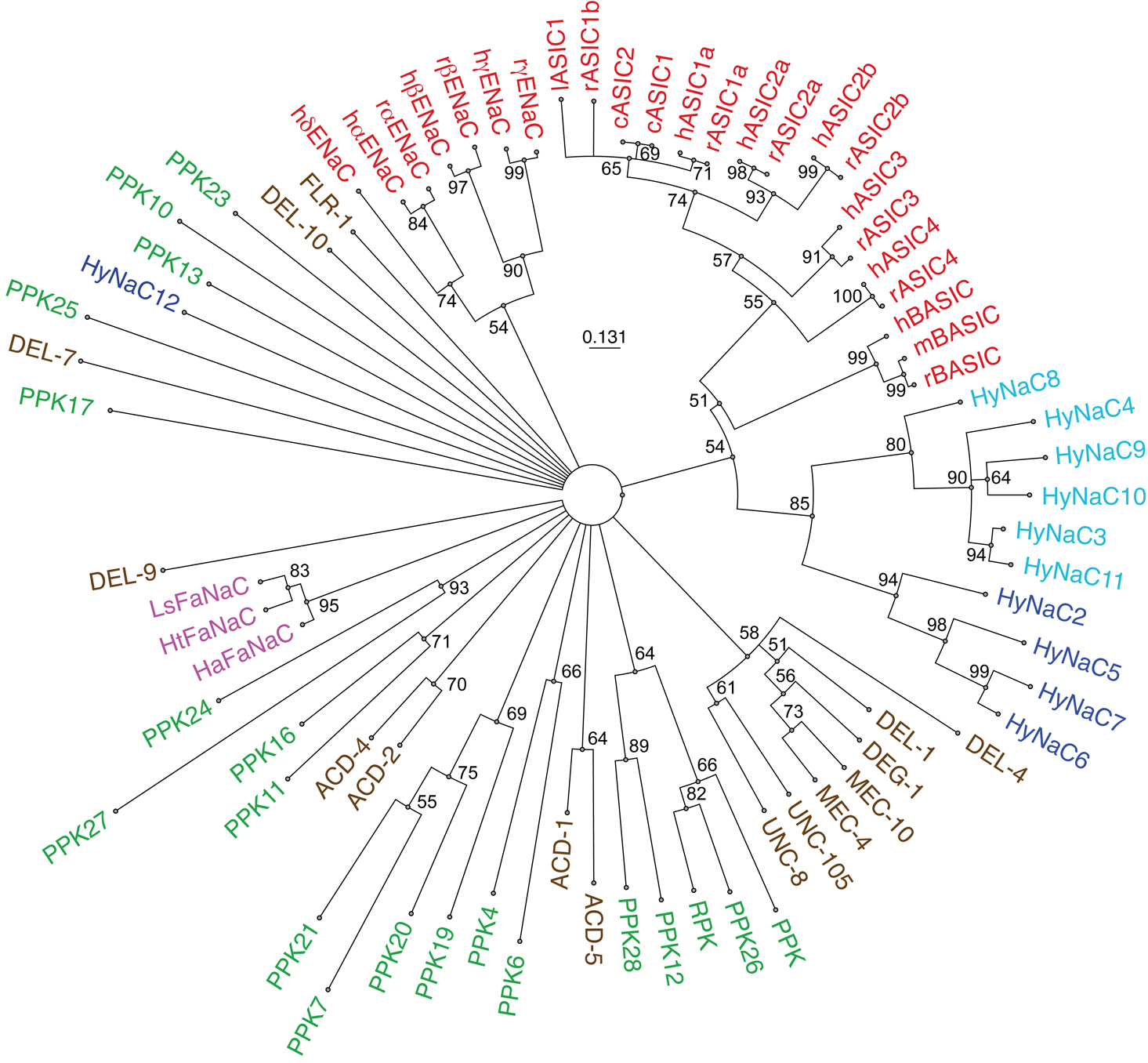

Supplement: Additional file 1 — Figure S1. Phylogenetic tree for the DEG/ENaC family generated by maximum likelihood analysis. The phylogenetic tree was generated by maximum likelihood analysis with PhyML (see Methods). Support values are indicated. Scale bar indicates amino acid exchanges per site. Color code, abbrevations of species names, and accession numbers are as in Figure 1. [file 12915_2014_84_MOESM1_ESM.jpeg]

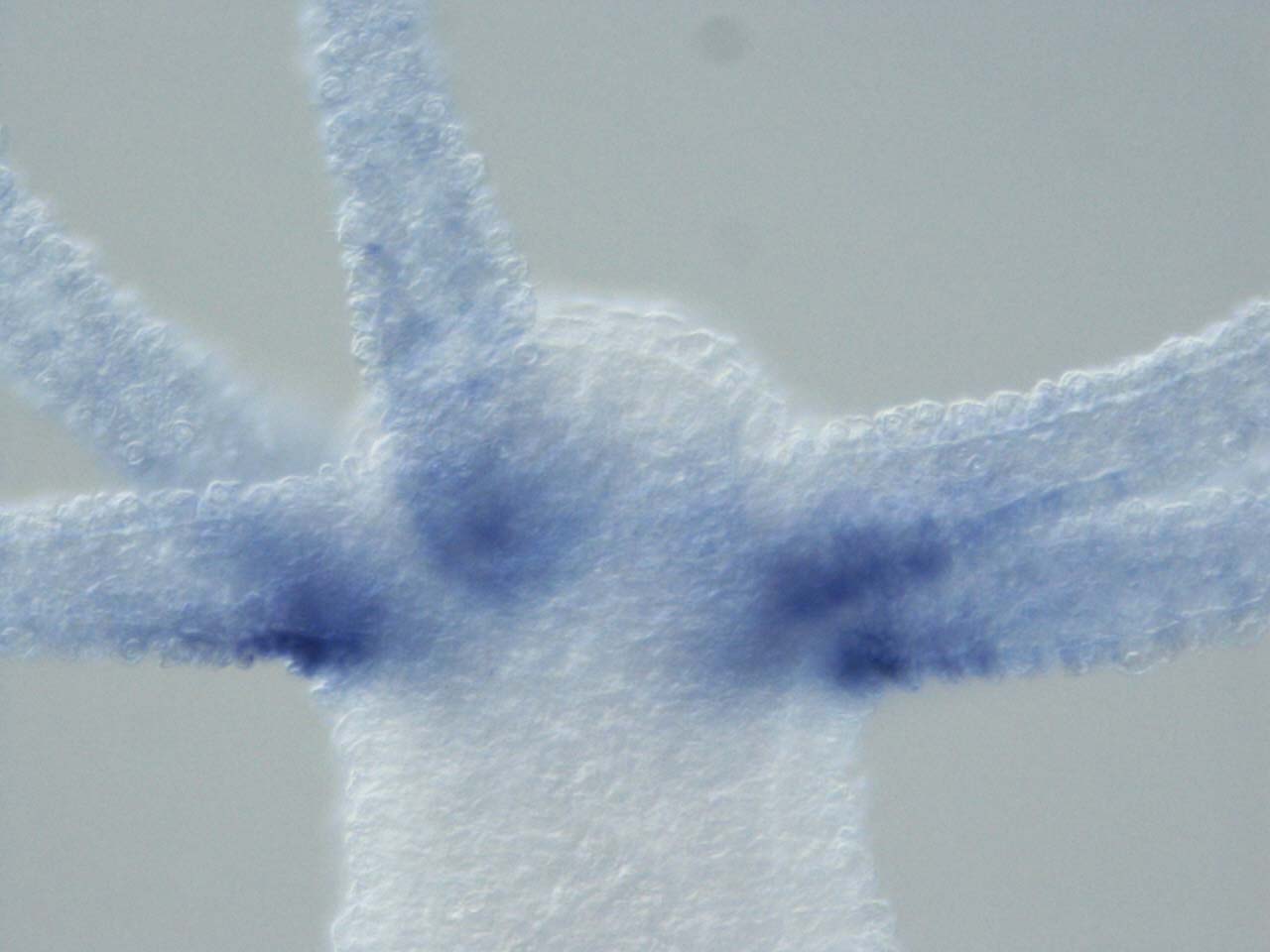

Supplement: Additional file 2 — Figure S2. High magnification of whole mount in situ hybridization for hynac4 revealing expression in epitheliomuscular cells at the base of the tentacles. [file 12915_2014_84_MOESM2_ESM.jpeg]
